# Regionalization of Habitat Suitability of Masson’s Pine based on geographic information system and Fuzzy Matter-Element Model

**DOI:** 10.1038/srep34716

**Published:** 2016-10-03

**Authors:** Xiuteng Zhou, Manxi Zhao, Liangyun Zhou, Guang Yang, Luqi Huang, Cuiqi Yan, Quanshu Huang, Liang Ye, Xiaobo Zhang, Lanpin Guo, Xiao Ke, Jiao Guo

**Affiliations:** 1 State Key Laboratory of Dao-di Herbs, National Resource Center for Chinese Materia Medica, China Academy of Chinese Medical Sciences, Beijing 100700, People’s Republic of China; 2 The Laboratory of Innovative Drug Development and Translational Medicine Research of Traditional Chinese Medicine, Chengdu Kanghong Pharmaceutical Limited Company, Chengdu 610036, People’s Republic of China; 3 Guangdong TCM Key Laboratory for Metabolic Diseases, Guangzhou 510006, People’s Republic of China; 4 Guangdong Pharmaceutical University, Guangzhou 510006, People’s Republic of China

## Abstract

Pine needles have been widely used in the development of anti-hypertensive and anti-hyperlipidemic agents and health food. However, the widespread distribution of this tree poses great obstacles to the quality control and efficacy evaluation. To facilitate the effective and rational exploitation of Masson’s pine (*Pinus massoniana* Lamb), as well as ensure effective development of Masson’s pine needles as a medicinal agent, we investigated the spatial distribution of habitat suitability and evaluated the optimal ranges of ecological factors of *P. massoniana* with 280 samples collected from 12 provinces in China through the evaluation of four constituents known to be effective medicinally. The results of habitat suitability evaluation were also verified by Root Mean Square Error (RMSE). Finally, five ecological factors were chosen in the establishment of a habitat suitability evaluation system. The most suitable areas for *P. massoniana* growth were mainly concentrated in the middle and lower reaches of the Yangtze River basin, such as Sichuan, Guizhou, and Jiangxi provinces, while the best quality needles were from Guizhou, Sichuan, and the junction area of Chongqing, Hunan, and Hubei provinces. This information revealed that suitable areas for effective constituent accumulation of Masson’s pine needles accounted for only 7.41% of its distribution area.

Masson’s pine needle, leaf of Masson’s pine (*Pinus massoniana* Lamb), in the family Pinaceae, is a traditional herbal medicine first recorded in Collective Notes to the Canon of Materia Medica^1^. It is known in Chinese medicine as a medicinal to dispel wind and dry dampness, kill parasites and relieve itching, calm the mind, and quicken the blood. Modern pharmacological research indicates that Masson’s pine needle extract has multiple biological activities including anti-hypertensive, anti-oxidant, anti-inflammatory, anti-tumor, anti-mutagenic, and hepatoprotective^2^. Research of its medicinal value is fairly well developed and the chemical composition of Masson’s pine needle has drawn wide attention^3^,^4^,^5^,^6^. Potential effective constituents of Masson’s pine needle were preliminarily selected based on previous research and pharmacological reports, including: (1) shikimic acid which exhibits multiple pharmacological effects, such as anti-bacterial, anti-inflammatory, anti-viral, and thrombolytic, and can effectively alleviate hypertension and inhibit platelet aggregation^7^,^8^; (2) procyanidins know as highly potent anti-oxidants and free radical scavengers, their anti-aggregation effect have been verified *in vivo*^9^; (3) flavonoids that inhibit oxidative injuries of the membranes of red blood cells, tissues, and organs including liver, kidney, spleen, and brain, hepatic mitochondria and microsomal, with effects on anti-lipid oxidation, anti-aging, and cellular activation of the brain and other organs^10^,^11^; (4) lignans with pharmacological effects including anti-tumor, anti-oxidant, anti-inflammatory, and *in vivo* inhibitory effects of platelet aggregation^12^,^13^,^14^.

*P. massoniana* is widely distributed in Jiangsu, Anhui, Henan, the Hanjiang river basin in Shaanxi, the middle and lower reaches of the Yangtze River, southern Fujian, Guangdong, Taiwan’s northern mountains and west coast, the eastern slopes of Daxiangling in central Sichuan, Guiyang and Bijie in Guizhou, and Funing in Yunnan, etc. Previous studies were limited to chemistry, or pharmacology and pharmacodynamics research, but areas suitable both for the growth and high medical quality of *P. massoniana* have not been evaluated. The vast difference between the climate, topography, and soils of different regions^15^ are likely to affect the quality of Masson’s pine needles.

Specific species habitat suitability and potential distribution areas are predicted by comparing the known target species distribution areas with the target habitat to find the suitable areas of the target species. This was done by using mathematical induction method and a simulated demand of its niche. In recent years, the maximum entropy model has been widely and successfully used in the prediction of suitable areas for crops, and potential habitat of medical plants^16^,^17^,^18^. However the maximum entropy model, focuses on the prediction of species distribution, but was not designed to predict the quality of the target species in different geographic environments. Using the Fuzzy matter-element model we solved this by quantifying the ambiguity, and the geographic information system (GIS) to support the space environment data. The relationship of plants and environment could be quantitatively investigated using the maximum entropy model analysis, Fuzzy function, and the GIS supported prediction of plant attributes space structure.

This study analyzed growth districts, quality division, and comprehensive regionalization of *P. massoniana* based on the climate characteristics of its primary producing areas, combined with contents of potentially effective components detected by HPLC. We also obtained spatial distribution of *P. massoniana* habitat suitability and the suitable range of each ecological factor. These data provided a theoretical basis for the protection and sustainable harvest of wild *P. massoniana*, as well as cultivation planning.

## Results

### Results prediction by maximum entropy model

Five ecological factors that affect the growth of *P. massoniana*, including four climatic and one topographical, were selected out of 55 candidates based on the maximum entropy model for the 280 samples, specifically precipitation in April and June, average atmospheric temperature for February and August, and altitude. The results showed that soil had little effect on *P. massoniana* growth, while climatic factors had a significant effect. AUC values of the training data set and test data set were both higher than 0.9, suggesting good simulations.

The optimal ranges of ecological factors according to each response curve were 98.8–112.8 mm for precipitation during April and 149.2–194.2 mm during June, 5–8 °C for average atmospheric temperature during February and 22–24 °C during August, and an altitude of 800–1100 meters.

**Figure 1:**
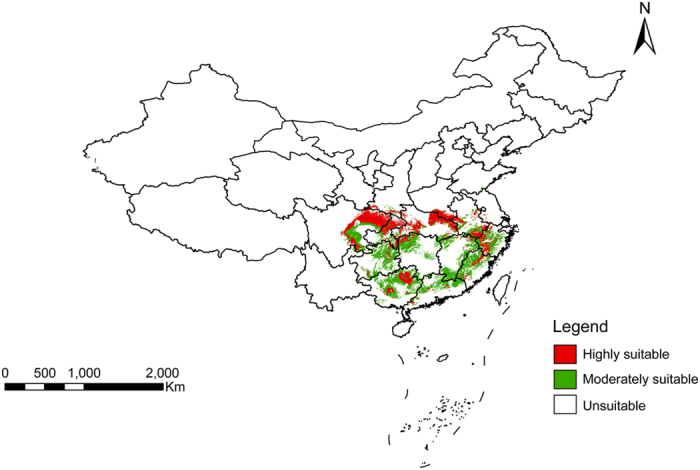
Results of Ecological Suitability Analysis for *P. massoniana*, Fig. 1 was based on prediction of maximum entropy model using Maxent software for species habitat modeling (version 3.3.3 k) (http://www.cs.princeton.edu/~schapire/maxent/), and superposition of those predicted spatial map layers using ArcMap 10.0 of the ArcGIS software, the URL for ArcGIS software purchase is http://www.esrichina.com.cn/, and the software version number is 10.0.

The results of the ecological suitability analysis for *P. massoniana* showed areas suitable for *P. massoniana* growth mainly concentrated in the middle and lower reaches of the Yangtze River basin, especially Sichuan, Guizhou, and Jiangxi provinces, as shown in Fig. 1.

### Establishment of Fuzzy matter-element model

Contents of four effective compounds or groups of compounds, each considered to have equal importance because they have significant pharmacological effects, were used as an aggregative indicator to analyze the evaluated environmental factors. Ranges of suitability values for each factor were solved by threshold calculation using MATLAB. Each evaluated environmental factor was standardized by threshold values and membership function, as shown in Table 1.

### Results of weighting of evaluation factors and distribution divisions allowing for the aggregative indicator

Contributions of the evaluated ecological factors, as shown in Table 2, revealed that climatic and topographic factors contributed 87% and 13%, respectively, suggesting that climatic factors had greater effects on the aggregative indicators in *P. massoniana*. And, within the 4 climatic factors, contribution of temperature (monthly average temperature February and August combined) was 69.7%, much higher than precipitation (precipitation in April and June combined), which was 17.3%, suggesting temperature is the most important factor in accumulation of the aggregative indicators in *P. massoniana*.

**Figure 2:**
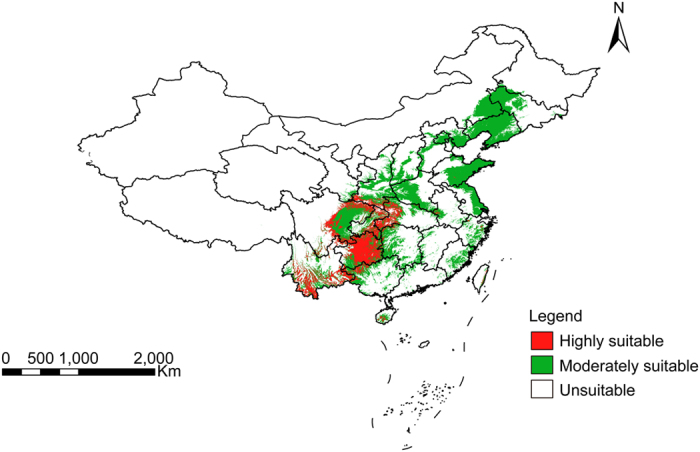
Result of Quality Suitability Analysis for *P. massoniana* Fig. 2 is based on the spatial interpolation of chemical components and layer superposition using ArcMap 10.0 in ArcGIS, the URL for ArcGIS software purchase is http://www.esrichina.com.cn/, and the software version number is 10.0.

**Figure 3:**
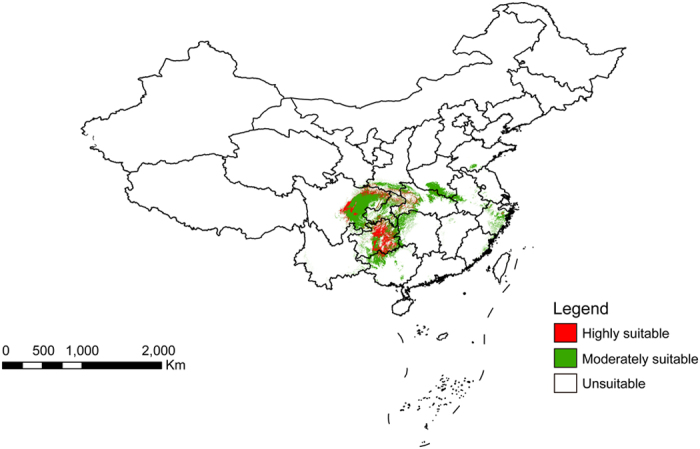
Result of Production suitability Analysis for *P. massoniana*, Fig. 3 is the result of spatial overlay of Figs 1 and 2, the URL for ArcGIS software purchase is http://www.esrichina.com.cn/, and the software version number is 10.0.

The results of the quality suitability analysis, as shown in Fig. 2, revealed areas for high quality Masson’s pine needles are mainly concentrated in Guizhou, Sichuan, and the intersection of Chongqing, Hunan, and Hubei provinces. Production suitability division was produced by overlaying layers of growth suitability, quality suitability, and land-cover types for the utilization and cultivation of *P. massoniana*, as shown in Fig. 3. The results showed the area and distribution of highly suitable, marginally suitable, and unsuitable habitat for *P. massoniana* production, and that the area of suitable habitat allowing for the aggregative indicator (sum of suitable and marginally suitable habitat) accounts for only 7.41% of the area where *P. massoniana* is distributed in China, as shown in Table 3.

### Result of model accuracy test

The RMSE value of the model for habitat suitability analysis was 0.1329, indicating feasibility of the model.

## Discussion

*P. massoniana* is widely distributed in sub-tropical areas in China. Its northern range is from Henan and the southern part of Shandong to Guangdong, Guangxi, and Taiwan in the south. It is found throughout eastern coastal areas of China to central Sichuan and Guizhou, and is abundant in middle and lower reaches of Yangtze River^19^,^20^,^21^. Masson’s pine needle is a traditional Chinese medicine, and its pharmacological effects have a significant body of literature supporting many traditional clinical applications^2^. However, the effects of ecological factors on the growth and quality of Masson’s pine needle have not been previously analyzed. In this study, the Maxent, Fuzzy matter element model, and GIS technology were combined to evaluate the ecological suitability, quality division, and production division of *P. massoniana*^18^,^22^. Results showed that the AUC value of the maximum entropy model was higher than 0.9, the RMSE value of the Fuzzy matter element model was 0.1329, indicating that the model was appropriate for use. The results of this research revealed that the area of suitable habitat (sum of suitable and marginally suitable habitat) accounts for only 7.41% of the area where *P. massoniana* is currently distributed in China, with areas in Guizhou and Sichuan provinces being some of the most suitable habitats. The prediction results also suggested the area suitable both for *P. massoniana* growth and high-quality needle production was not large. Therefore, with the development of Masson’s pine needles for medical use, along with use of the wood for other economically valuable products, it appears to be problematic to ensure the sustainable utilization of *P. massoniana*.

Newly emerging studies on habitat suitability analysis and quality prediction under different geographical conditions for medicinal plants mostly use one method, such as the mathematical method, model of induction, and maximum entropy model for analysis. This study was the first to combine several methods effectively to investigate the spatial distribution of habitat suitability, avoided the inherent problems of using a single method, and the result can provide a reference for the selection of suitable areas for *P. massoniana* cultivation.

It has been suggested that for the sustainable utilization of *P. massoniana*, nature reserves in suitable habitats should be established^23^,^24^,^25^. Moreover, cultivation is an essential method to enlarge the population and increase yield by selecting high quality breeds and improving cultivation methods^26^.

## Methods

### Data acquisition

Masson’s pine needles were collected from all 12 provinces where *P. massoniana* is distributed in China, including the provinces of: Guizhou, Hunan, Guangxi, Fujian, Hubei, Guangdong, Sichuan, Zhejiang, Henan, Jiangxi, Chongqing, and Anhui.

**Figure 4:**
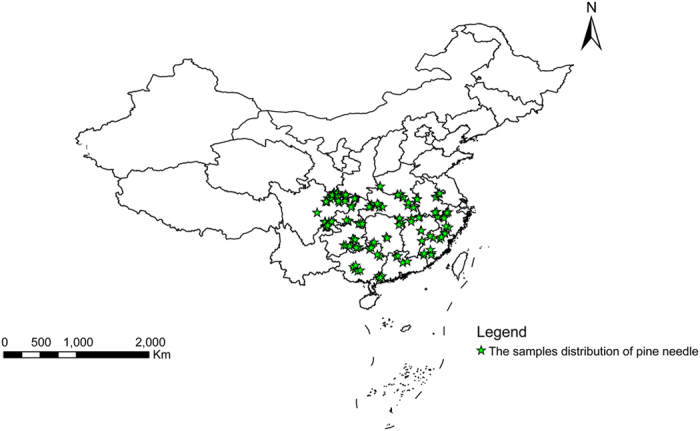
Sampling Distribution of Pine, Fig. 4 is based on the data loading of longitude and latitude data of sampling points using ArcMap 10.0, the URL for ArcGIS software purchase is http://www.esrichina.com.cn/, and the software version number is 10.0.

On site sampling was conducted at 140 sites and 280 fresh pine needle samples were collected, the sampling distribution is shown in Fig. 4. Geographic information including altitude, longitude, and latitude was recorded by GPS. Contents of shikimic acid, procyanidins, total flavonoids, and total lignans were quantitatively determined by HPLC, as shown in “Supplementary Table S1”.

### Selection of evaluation parameters

Climate, soils, topography, and vegetative covers are 4 major classes of ecological factors that influence plant growth^27^,^28^. A total of 55 ecological factors (shown in “Supplementary Table S2”) recorded in the “Online Database of Spatial Distribution Information of Chinese Medicine Resources” (http://www.tcm-resources.com/) were selected as candidates including 43 climate factors (monthly precipitation, monthly average temperature, etc.), 8 soil factors, 3 topographic factors (altitude, slope, and aspect), and vegetative covers. The most influential factors were chosen out of 55 ecological factors using a maximum entropy model for the establishment of habitat suitability evaluation system.

### Fuzzy matter-element model

In order to decide which functions to use, function selection was based on the specific relationship between the contents of constituents and ecological factors; characters of the subject and the functions; and first curve fitting between the contents of constituents. Finally, values in the functions were worked out with MATLAB. Using this method, the content of constituents and the ecological factors were established. Then a suitability value ranging from 0 to 1 was defined for each ecological factor based on function. In a Fuzzy set, 0 means a certain element has absolutely no function to a Fuzzy set (unsuitable), 1 means a certain element completely belongs to a Fuzzy set (entirely suitable). 0 means the ecological factor is completely unsuitable for its growth, while 1 means the ecological factor is completely suitable for its growth, and a value between 0 and 1 represents partial suitability^29^,^30^,^31^,^32^.

The selection of functions and parameter estimates of the ecological factors were conducted by a curve fitting from 5 available candidates shown below (1–5). The ecological factors were standardized by choosing K-t function (i.e. function 1) according to the curve fitting^33^.


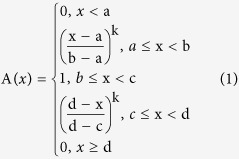



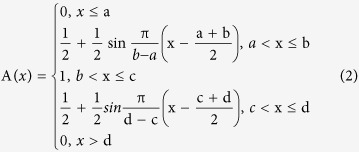



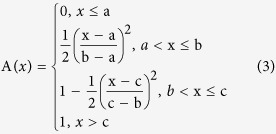



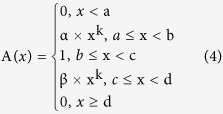



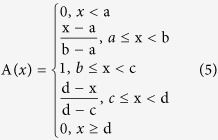


a, b, c, d, α, β and K represent the a, b, c, d, α, β and K in K-t function, respectively.

### Weighting of evaluation factors

Habitat suitability can be evaluated by many approaches, e.g. analytic hierarchy process^34^, logistic regression^35^, etc. Comparing these methods, maximum entropy model requires a smaller sample size^36^. Moreover, though accuracy of model output depends on the quantity of observation data, the maximum entropy model is capable of yielding a correct solution based on fewer observation data^37^. There is little published research on habitat suitability of *P. massoniana*, thus expertise in this subject is insufficient. Therefore, weighing coefficients were evaluated by the maximum entropy model in objective weight method.

### Model accuracy

Using RMSE to test the results of habitat suitability evaluation^30^,^38^,^39^,^40^,^41^.

### Output divisions

Based on weighing of each evaluation factor, diagram layers of evaluation factors were calculated by a weighted mean and grid computing in ArcGIS, grid size was set as the maxim value of input grids. A spatial distribution diagram of habitat suitability with grid size of 1 km × 1 km was generated by computation. The superposition of the diagram and the coniferous forest cover type was made in order to eliminate unsuitable land-cover types including farmland, rivers, lakes, urban ares, etc., and to yield the final version of spatial distribution diagram of habitat suitability^22^.

## Additional Information

**How to cite this article**: Zhou, X. *et al*. Regionalization of Habitat Suitability of Masson’s Pine based on geographic information system and Fuzzy Matter-Element Model. *Sci. Rep.* **6**, 34716; doi: 10.1038/srep34716 (2016).

## Supplementary Material

Supplementary Information

Supplementary Dataset 1

## Figures and Tables

**Table 1:** Results of Fuzzy Membership Functions on The Evaluated Environment Factors.

	a	b	c	d	K*
Precipitation in April (mm)	−63.9	98.8	113.8	191.3	1.62
Precipitation in June (mm)	−63.7	149.2	194.2	427.4	2.51
Monthly avg. in February (°C)	−42.4	5	8	33.6	13
Monthly avg. in August (°C)	20	22	24	36.1	2.93
Altitude (m)	−900	800	1181	2195	2.9

^*^a, b, c, d and K represent the a, b, c, d and K in K-t membership function in 2.3, respectively.

**Table 2:** Contribution of Individual Variable to Habitat Suitability Allowing for the Aggregative Indicator.

Type	Variable	Percent contribution
Climatic (87.0%)	Precipitation in April	3.4%
	Precipitation in June	13.9%
	Monthly avg. for February	25.8%
	Monthly avg. for August	43.9%
Topographical (13.0%)	Altitude	13.0%

**Table 3:** Areas and Distribution of Highly Suitable, Marginally Suitable, and Unsuitable Habitats.

Highly class	Area and percentage	Distributions of province and municipality
Highly suitable habitat	14769.86 m^2^ (1.69%)	Jiangsu, Zhejiang, Anhui, Henan, Hubei, Hunan, Guangxi, Chongqing, Sichuan, Guizhou, Yunnan, Shaanxi
Marginally suitable habitat	49966.0986 m^2^ (5.72%)	Jiangsu, Zhejiang, Anhui, Fujian, Jiangxi, Shandong, Henan, Hubei, Hunan, Guangxi, Chongqing, Sichuan, Guizhou, Yunnan, Shaanxi
Unsuitable habitat	809140.01 m^2^ (92.59%)	All province and municipality
