# Supplementary material for: Regionalization of Habitat Suitability of Masson’s Pine based on geographic information system and Fuzzy Matter-Element Model
Source: Sci Rep. 2016 Oct 3;6:34716. doi: 10.1038/srep34716 (PMC5046140; doi:10.1038/srep34716)
Supplement: Supplementary Information [file srep34716-s1.pdf]

Regionalization of Habitat Suitability of Masson’s Pine based on geographic information system and Fuzzy Matter-Element Model

Xiuteng Zhou[1+], Manxi Zhao[2+], Liangyun Zhou[1+], Guang Yang[1], Luqi Huang[1], Cuiqi Yan[2],
Quanshu Huang[2], Liang Ye[2], Xiaobo Zhang[1], Lanpin Guo[1*],
Xiao Ke[2*], Jiao Guo[3, 4*]

Table S1 Origins and contents of 280 samples from *Pinus massoniana* Lamb.

Sites No.	Sample No.	Longitude	Altitude	Samlpe localtion	Shikimic acid (mg/g)	Procyanidins (mg/g)	Flavonoids (mg/g)	Lignans (mg/g)
1	1	104.6448°	32.0018 °	Bajiao Village, Guixi Township, Beichuan County, Mianyang City, Sichuan Province	0.43324082	1.2589416	0.00345800	0.52166919
	2				0.43919552	1.2392625	0.00339220	0.52226153
2	3	105.1762 °	32.2087 °	Yanmen Township, Jiangyou County-level City, Mianyang City, Sichuan Province	0.40519033	0.9195982	0.00237532	0.59610385
	4				0.40702411	0.9308159	0.00257661	0.59487627
3	5	105.0029 °	31.8997 °	Xiaoxi dam Township,Jiangyou County-level City, Mianyang City, Sichuan Province	0.50028985	1.0683837	0.00285348	0.55529261
	6				0.51168040	1.0633398	0.00309980	0.58891215
4	7	104.3775 °	31.5499 °	Red Village, Xiushui Township, Anxian County, Mianyang City, Sichuan Province	0.50344273	1.1652186	0.00307112	0.62767998
	8				0.49905448	1.1697137	0.00293539	0.62239099
5	9	104.5879 °	31.5578 °	Baiyang Village, Flowers Gai Township, Anxian County, Mianyang City, Sichuan Province	0.51863907	1.0919303	0.00292441	0.71318746
	10				0.51145677	1.0857161	0.00310964	0.67704635
6	11	104.4667 °	31.5732 °	The bodhi Village, HuangTu Township, Anxian County, Mianyang City, Sichuan Province	0.47262853	1.017678	0.00323980	0.67186809
	12				0.47428038	1.0756056	0.00321497	0.68092611
7	13	103.3513 °	30.2185 °	Maohe Village, Maohe Township, Famous mountains Area, Yaan City, Sichuan Province	0.41765500	0.9112142	0.00412878	0.60728143
	14				0.42343711	0.9382795	0.00375593	0.60107029
8	15	103.3526 °	30.1941 °	Xuyuan Village, Lianjiang Township, Famous mountains Area, Yaan City, Sichuan Province	0.47599068	0.8466142	0.00396400	0.62979673
	16				0.47750081	0.8373119	0.00395503	0.62811462
9	17	105.3832 °	32.6357 °	Dam on border Communities, Slab bridge Village, MuYu Township, Qingchuan County	0.42651087	0.9738755	0.00349953	0.86243249

9	18	[illegible]	[illegible]	[illegible] Village, [illegible] Township, Qingchuan County, Guangyuan City, Sichuan Province	0.43195194	1.0154085	0.00331793	0.87043217
10	19	105.4658 °	32.6742 °	Sandui Village, Shazhou Township, Qingchuan County, Guangyuan City, Sichuan Province	0.53020843	1.2384888	0.00234614	0.68912542
	20				0.53244473	1.221646	0.00247557	0.67759284
11	21	105.2317 °	32.3827 °	Liangsheng Village, Cold water Township, Qingchuan County, Guangyuan City, Sichuan Province	0.54268013	0.882027	0.00221955	0.64214683
	22				0.53933007	0.8777325	0.00234444	0.65129010
12	23	105.947 °	32.6694 °	Longmen Village, xuanhe Township, Chaotian District, guangyuan City, Sichuan Province	0.43546637	1.1438097	0.00348536	0.64029300
	24				0.43735393	1.2086672	0.00338296	0.65151347
13	25	105.806 °	32.6024 °	Wuba Village, Yangmu Township, Chaotian District, Guangyuan City, Sichuan Province	0.48719593	1.1813807	0.00300291	0.64470457
	26				0.50511372	1.1640377	0.00291172	0.64283660
14	27	105.8698 °	32.6588 °	Jindui Village, Chaotian Township, Chaotian District, Guangyuan City, Sichuan Province	0.38595428	1.7004	0.00271984	0.70227660
	28				0.38840045	1.5581719	0.00294256	0.70096503
15	29	105.9673 °	32.0368 °	Dangyang Village, Wencun Township, Zhaohua District, Guangyuan City, Sichuan Province	0.46536962	1.4898024	0.00260923	0.63216831
	30				0.47659300	1.544424	0.00257098	0.64735034
16	31	105.9534 °	32.3227 °	Osmanthus Village, Yuanba Township, Zhaohua District, Guangyuan City, Sichuan Province	0.48689214	1.3761923	0.00297902	0.74432862
	32				0.49628122	1.3913005	0.00366025	0.74313242
17	33	105.9641 °	32.1426 °	Gengxn Village, Wangjia Township, Zhaohua District, Guangyuan City, Sichuan Province	0.63053798	1.1236448	0.00307148	0.67152777
	34				0.62882967	1.14563	0.00331450	0.66932065
18	35	105.0947 °	29.0812 °	Tsing Shan Village, WanShou Township, FuShun County, Zigong City, Sichuan Province	0.46370254	0.5399927	0.00273029	0.49222290
	36				0.46072035	0.591473	0.00255591	0.49096856
19	37	104.8139 °	29.0649 °	Chuanzhu Village, Liqiao Township, FuShun County, Zigong City, Sichuan Province	0.49139640	0.7946928	0.00311866	0.58627191
	38				0.50309424	0.7527688	0.00301824	0.59678965
20	39	105.1529 °	29.1046 °	Seven Group, moxiang Village, Tongshi Township, FuShun County, Zigong City, Sichuan Province	0.51515239	1.0967167	0.00251195	0.47038307
	40				0.51938075	1.0934396	0.00261480	0.46078728
21	41	104.3462 °	29.1028 °	zhouchang Village, guanyin Township, Yibin County, Yibin City, Sichuan Province	0.40842333	1.5478802	0.00330880	0.64693430
	42				0.40052911	1.5240813	0.00348237	0.65845562
22	43	104.2933 °	29.0191 °	Jiangan Village, Nixi Township, Yibin County,	0.45128245	1.2387372	0.00264879	0.60748573

22	44	[illegible]	[illegible]	Yibin City, Sichuan Province	0.44610807	1.2324906	0.00257294	0.60890099
23	45	104.3818 °	28.4164 °	Shahe Village, shuanglong Township, Yibin County, Yibin City, Sichuan Province	0.44582347	1.238994	0.00525966	0.50874116
	46				0.44136915	1.2795545	0.00565928	0.50508670
24	47	104.5608 °	28.5962 °	Baixiang Village, Laifu Township, High County, Mianyang City, Sichuan Province	0.44193297	1.2523902	0.00267262	0.62643265
	48				0.43377487	1.2143069	0.00262308	0.60653963
25	49	104.4967 °	28.3921 °	Shuidong Village, Qingfu Township, High County, Mianyang City, Sichuan Province	0.40516129	1.5931403	0.00382617	0.70888721
	50				0.40195087	1.5454369	0.00420069	0.70426555
26	51	104.6669 °	28.6225 °	Cotton Village, Yuejiang Township, High County, Mianyang City, Sichuan Province	0.41008761	0.4724201	0.00266814	0.49682509
	52				0.41926461	0.445246	0.00214457	0.48476796
27	53	106.0818 °	31.4379 °	Deyang Village, Hongshan Township, Langzhong County, Nanchong City, Sichuan Province	0.50433998	1.1374127	0.00241681	0.71402069
	54				0.48625547	1.1849873	0.00255676	0.70639596
28	55	105.9686 °	31.6462 °	Jingu Village, Stone Township, Langzhong County, Nanchong City, Sichuan Province	0.50521190	1.6265922	0.00265849	0.62048553
	56				0.49823640	1.6714981	0.00285344	0.61218181
29	57	106.9853 °	31.9761 °	Xiejiawan Street, Zitong temple Township, Bazhou District, Bazhong City, Sichuan Province	0.44083418	1.5223893	0.00330088	0.63372365
	58				0.44952193	1.5937383	0.00331308	0.64070277
30	59	106.8671 °	31.986 °	Shizizhai Village, Shiling Township, Bazhou District, Bazhong City, Sichuan Province	0.58962823	1.5087094	0.00268215	0.63818847
	60				0.57695946	1.5023009	0.00252353	0.63574043
31	61	106.7959 °	31.6129 °	Longbao Village, Dingshan Township, Bazhou District, Bazhong City, Sichuan Province	0.50741032	0.8289145	0.00281205	0.51602642
	62				0.50930279	0.8258023	0.00318713	0.51863509
32	63	106.787 °	32.2183 °	Tianshengqiao Village, Shahe Township, Nanjiang County, Bazhong City, Sichuan Province	0.28724949	0.6917023	0.00280052	0.57128732
	64				0.28574773	0.6339993	0.00283485	0.57081592
33	65	106.8405 °	32.3956 °	Peak Village, Liuba Township, Nanjiang County, Bazhong City, Sichuan Province	0.44769274	0.9863298	0.00194671	0.42050238
	66				0.43140910	0.9822211	0.00191322	0.40830655
34	67	106.8943 °	32.4179 °	Lujiaoya Village, Ganchang Township, Nanjiang County, Bazhong City, Sichuan Province	0.47069153	1.1065003	0.00251609	0.58044483
	68				0.48188220	1.1114533	0.00241357	0.57032276
35	69	107.6576 °	31.0699 °	Daqiaoer Village, Dafeng Township, Daxian County, Dazhou City, Sichuan Province	0.42872855	1.3294467	0.00385514	0.55325682
	70				0.42855982	1.3300913	0.00367629	0.55046334

36	71	107.6364°	31.0853°	Victory Village, Pavilion Township, Daxian County, Dazhou City, Sichuan Province	0.50899293	1.8279871	0.00356896	0.66185663
	72				0.51411434	1.8006541	0.00357844	0.66196614
37	73	107.5666°	30.8634°	Yinzi Village, Nanyue Township, Daxian County, Dazhou City, Sichuan Province	0.47804936	1.2127224	0.00316247	0.56110060
	74				0.48506026	1.2515128	0.00347708	0.56016170
38	75	108.1718°	31.9607°	Sumac ping Village, Baisha Township, Wanyuan County-level City, Dazhou City, Sichuan Province	0.45139067	1.2404289	0.00208950	0.47865457
	76				0.44927613	1.2318394	0.00237892	0.46718784
39	77	108.0757°	32.0459°	Lijiagou Village, Chaya Township, Wanyuan County-level City, Dazhou City, Sichuan Province	0.49299471	1.7808745	0.00341918	0.64665138
	78				0.49169374	1.7335894	0.00327827	0.64807133
40	79	108.0019°	31.9461°	Fangjialiang Village, Qinghua Township, Wanyuan County-level City, Dazhou City, Sichuan Province	0.51264707	0.2394707	0.00252547	0.43937780
	80				0.50647515	0.2305386	0.00307455	0.43796236
41	81	107.1882°	29.1321°	SanQuan Village, SanQuan Township, Nanchuan District, Chongqing Municipality	0.44660052	1.7503442	0.00337567	0.93358689
	82				0.44783033	1.7921291	0.00348220	0.94492321
42	83	107.2202°	29.2185°	Nanzhu mountain forest park, Nanchuan District, Chongqing Municipality	0.46454812	1.5904471	0.00249090	0.44608582
	84				0.46323672	1.7224968	0.00246240	0.43793521
43	85	106.9558°	29.2632°	Yuwu Village, Daguan Township, Beichuan County, Nanchuan District, Chongqing Municipality	0.48958357	1.1708053	0.00236677	0.57661312
	86				0.48441006	1.0952883	0.00244795	0.56443519
44	87	108.7205°	28.8602°	East LiuKou Village, Peach Garden Township, Qiuyang County, Chongqing Municipality	0.62627924	1.169285	0.00281551	1.15998670
	88				0.63640442	1.2231369	0.00329484	1.15169287
45	89	108.4952°	28.8179°	Taibai Village, Tianguan Township, Qiuyang County, Chongqing Municipality	0.54081987	0.9864038	0.00262088	0.56926879
	90				0.55737609	0.9775577	0.00268033	0.56480288
46	91	108.9538°	28.7905°	Meizhu Village, LongTan Township, Qiuyang County, Chongqing Municipality	0.55457765	1.5678581	0.00394813	0.72871302
	92				0.53544117	1.5943753	0.00389853	0.71807856
47	93	110.0931°	31.2091°	Mule ping Village, Mule ping Township, Wushan County, Chongqing Municipality	0.60428244	1.0506784	0.00312006	0.86389420
	94				0.59174009	1.0551549	0.00266170	0.87806646
48	95	110.0026°	30.9721°	Hejia Village, Baolong Township, Wushan County, Chongqing Municipality	0.49818753	1.0486525	0.00435828	1.14785054
	96				0.48854995	1.164237	0.00458017	1.14110303

49	97	109.8662 °	30.9822 °	Yangba Village, Guandu Township, Wushan County, Chongqing Municipality	0.54409785	1.4600029	0.00408479	0.86004628
	98				0.53972959	1.3765418	0.00401027	0.86417659
50	99	109.866 °	30.9821 °	Yangba Village, Guandu Township, Wushan County, Chongqing Municipality	0.75178375	1.1864006	0.00401495	0.71824150
	100				0.72619370	1.1649996	0.00400721	0.72974052
51	101	109.7882 °	30.9145 °	Longtai Village, Guandu Township, Wushan County, Chongqing Municipality	0.53081186	1.9268324	0.00512353	1.06434457
	102				0.55180528	1.9351444	0.00498392	1.08427799
52	103	106.6485 °	26.1746 °	Moguang Village, Peaceful Township, Huishui County, Buyi and Miao Autonomous Prefecture of QianNan, Guizhou Province	0.57887798	0.487199	0.00191806	0.64290789
	104				0.58088585	0.5428415	0.00185100	0.62654743
53	105	106.908 °	26.1639 °	BaiLi Village, Gangdu Township, Huishui County, Buyi and Miao Autonomous Prefecture of QianNan, Guizhou Province	0.54067386	0.840741	0.00273882	0.53936446
	106				0.54533008	0.8978847	0.00266540	0.54624439
54	107	107.6019 °	26.1768 °	Horse Village, Daping Township, Dujun City, Buyi and Miao Autonomous Prefecture of QianNan, Guizhou Province	0.57085399	0.960947	0.00195417	0.62052497
	108				0.57078353	0.9559052	0.00201324	0.61698031
55	109	107.6019 °	26.1768 °	Horse Village, Daping Township, Duyun City, Buyi and Miao Autonomous Prefecture of QianNan, Guizhou Province	0.53279473	1.5555274	0.00264944	0.56989569
	110				0.52787168	1.4666843	0.00272995	0.57416727
56	111	107.3768 °	26.0517 °	Flat forest farm, Flat forest Township, Duyun City, Buyi and Miao Autonomous Prefecture of QianNan, Guizhou Province	0.47809058	1.6562685	0.00201848	0.58275032
	112				0.47120897	1.6118671	0.00186850	0.59544914
57	113	108.5125 °	25.9509 °	Happy Village, Ancient Township, Rongjiang City, Qiandongnan Miao and Dong Autonomous Prefecture, Guizhou Province	0.49219510	1.0465265	0.00274275	0.57880454
	114				0.49389537	1.0288939	0.00246859	0.57718141
58	115	108.1775 °	25.8506 °	Gaojiu Village, Huashuizu Township, Rongjiang City, Qiandongnan Miao and Dong Autonomous Prefecture, Guizhou Province	0.37900980	1.3778704	0.00312532	0.76968801
	116				0.38403589	1.424194	0.00336984	0.78549740
59	117	107.8961 °	26.9182 °	Wotian Village, Xinzhou Township, Huangping City, Qiandongnan Miao and Dong Autonomous Prefecture, Guizhou Province	0.50706781	1.0312436	0.00436580	1.18396145
	118				0.51726742	1.035225	0.00416640	1.18276744
60	119	107.7584 °	26.9537 °	Pingxiba Village, Jiuzhou Township, Huangping City, Qiandongnan Miao and Dong Autonomous Prefecture, Guizhou Province	0.51195147	1.1432117	0.00297775	0.58052171
	120				0.50996234	1.1015122	0.00273613	0.58399895
61	121	108.0627 °	26.858 °	Pond Village, Gulong Township, Huangping City, Qiandongnan Miao and Dong Autonomous Prefecture, Guizhou Province	0.51034307	1.3402647	0.00237520	0.48139357
	122				0.51330030	1.4099882	0.00262504	0.48177803
62	123	111.9064 °	27.1932 °	Feng tree Village,Fire factory ping Township, Shaodong County, Shaoyang City, Hunan	0.51746614	1.0735461	0.00273581	0.86626679

62	124	111.9064	27.1932	Shaodong County, Shaoyang City, Hunan Province	0.51819410	1.1178361	0.00254867	0.86301037
63	125	112.0279 °	27.0414 °	Stone Village, Front of fort Township, Shaodong County, Shaoyang City, Hunan Province	0.50221272	1.5068045	0.00211951	1.20315561
	126				0.49604295	1.4909084	0.00226615	1.19707662
64	127	110.147 °	26.3379 °	Shangpai Village, Changan camp Township, Chengbu County, Shaoyang City, Hunan Province	0.46059021	0.7485978	0.00187452	0.74191316
	128				0.46217690	0.7420774	0.00197129	0.74335400
65	129	110.3873 °	26.5273 °	Datang Village, Maoping Township, Chengbu County, Shaoyang City, Hunan Province	0.47767450	1.0402149	0.00313692	0.68905666
	130				0.47482637	0.9827783	0.00346418	0.70035123
66	131	110.3873 °	26.5273 °	Datang Village, Maoping Township, Chengbu County, Shaoyang City, Hunan Province	0.46590844	0.4550152	0.00182980	0.65583507
	132				0.46533396	0.4832698	0.00165828	0.65071719
67	133	113.4976 °	29.3114 °	Dongchong Village, Zhanqiao Township, Linxiang City, Hunan Province	0.39044321	0.4823499	0.00288519	0.49410063
	134				0.39160206	0.4846221	0.00254332	0.49941539
68	135	113.5578 °	29.5071 °	Luoxing Village, Lousi Township, Linxiang City, Hunan Province	0.44300587	1.0598833	0.00298434	0.78704276
	136				0.44650667	1.0601159	0.00280395	0.79466015
69	137	113.8067 °	28.8529 °	Taoxia Village, Hongqiao Township, Pingjiang County, Yueyang City, Hunan Province	0.39823339	1.1516499	0.00250106	0.48095483
	138				0.40574628	1.1450479	0.00254686	0.48682699
70	139	113.4762 °	28.6954 °	Small pond Village, Wengjiang Township, Pingjiang County, Yueyang City, Hunan Province	0.44886899	1.1377654	0.00256512	0.55065625
	140				0.45568124	1.1914851	0.00253609	0.54456869
71	141	109.8378 °	25.6557 °	Shuanglang Village, Sanmen Township, Longsheng County, Guilin City, Guangxi Province	0.42834127	0.475462	0.00456235	0.85906194
	142				0.41417188	0.4764131	0.00457168	0.85230366
72	143	110.0451 °	25.8544 °	Liwucun Village, Sishui Township, Longsheng County, Guilin City, Guangxi Province	0.61363208	0.5945411	0.00259286	0.58866685
	144				0.61359002	0.6016502	0.00266239	0.58860799
73	145	111.0907 °	24.7665 °	Anchong Village, Sanjiang Township, Gongcheng County, Guilin City, Guangxi Province	0.46616412	1.1383005	0.00242356	0.71170418
	146				0.47866239	1.1673789	0.00261608	0.72105457
74	147	110.818 °	25.0188 °	Sue vicious Village, Jiahui Township, Gongcheng County, Guilin City, Guangxi Province	0.43228958	1.2861494	0.00382783	0.60517335
	148				0.43347641	1.2843524	0.00261994	0.60555085
75	149	108.0007 °	23.1721 °	Gong dongshanwei Township, Wuming County,	0.44755976	1.0755886	0.00452153	0.70594491

75	150	[illegible]	23.1721	Nanning City, Guangxi Province	0.42979686	1.0653827	0.00466204	0.71253271
76	151	107.835 °	23.4718 °	Yongchuan forest farm, Yongchuan Township, Mashan County, Nanning City, Guangxi Province	0.40579698	0.4272598	0.00188591	0.35955372
	152				0.41314829	0.3991951	0.00174891	0.36684669
77	153	108.1192 °	23.6723 °	Songjiang Village, Jolly Township, Mashan County, Nanning City, Guangxi Province	0.47590587	1.3848322	0.00310875	0.52948695
	154				0.46178885	1.3503718	0.00315465	0.52354637
78	155	108.3788 °	22.9705 °	Dongsheng parvial field, Peak forest farm, Xingning County, Nanning City, Guangxi Province	0.38701535	0.6490044	0.00161490	0.46270980
	156				0.39177034	0.6444799	0.00179576	0.46373276
79	157	108.6792 °	23.0618 °	Jiutang Village, Kunlun Township, Xingning County, Nanning City, Guangxi Province	0.48206083	1.536265	0.00246937	0.44950425
	158				0.46296383	1.5007693	0.00247412	0.45054630
80	159	119.5897 °	30.2802 °	Fanlong Village, Taihuyuan Township, Linan County, Hangzhou City, Zhejiang Province	0.43288345	1.1727136	0.00205372	0.50573523
	160				0.43375880	1.186639	0.00223251	0.50513002
81	161	119.4197 °	30.1823 °	Mingfeng Village, Yuqian Township, Linan County, Hangzhou City, Zhejiang Province	0.46513287	0.9178099	0.00183879	0.50953307
	162				0.46673003	0.8808233	0.00193872	0.51265684
82	163	118.9432 °	29.681 °	Shanhou Village, Jinfeng Township, Chunan County, Hangzhou City, Zhejiang Province	0.56257917	1.3643862	0.00383023	0.74537877
	164				0.57223070	1.3275471	0.00416184	0.75390971
83	165	119.0017 °	29.7031 °	The eastern han dynasty Village, Left mouth Township, chunan County, Hangzhou City, Zhejiang Province	0.49641618	1.635408	0.00375242	0.90000580
	166				0.49289150	1.62703	0.00427430	0.89466590
84	167	119.0539 °	29.5186 °	LaoShanDao, Chunan County, Hangzhou City, Zhejiang Province	0.46844458	1.1823817	0.00359503	0.81223262
	168				0.46885394	1.1760557	0.00485779	0.80403514
85	169	119.1645 °	27.7215 °	Chegeng Village, Baishanzhu Township, Qingyuan County, Lishui City, Zhejiang Province	0.46558343	0.9529515	0.00215844	0.99176101
	170				0.46589555	0.911128	0.00213115	0.99382245
86	171	119.1742 °	27.6081 °	Yanxia Village, Wudabao Township, Qingyuan County, Lishui City, Zhejiang Province	0.38235469	1.0705128	0.00355621	0.73273205
	172				0.38837606	1.0772317	0.00354619	0.73583244
87	173	119.6058 °	28.3689 °	Xiangxi Village, Xiangxi Township, Songyang County, Lishui City, Zhejiang Province	0.31563462	1.5841021	0.00539573	0.94818878
	174				0.31642848	1.5929403	0.00541163	0.95688209
88	175	119.2974 °	28.5116 °	Dalinggeng Village, Xinxing Township, Songyang County, Lishui City, Zhejiang Province	0.41303131	1.024821	0.00337854	0.85877979
	176				0.41530602	1.0195788	0.00343679	0.85331096

89	177	118.2983 °	29.6567 °	Linxi Village, Donglinxi Township, TunXi District, Huangshan City, Anhwei Province	0.40505571	1.0121378	0.00250573	0.74818641
	178				0.40829803	1.0292739	0.00280198	0.75344999
90	179	118.2619 °	29.7047 °	Jianzhong Village, Liyang Township, TunXi District, Huangshan City, Anhwei Province	0.43937957	0.8374269	0.00315306	0.52582534
	180				0.44097517	0.8391021	0.00307170	0.52572016
91	181	118.1364 °	30.3279 °	Majia Village, Gantang Township, Huangshan District, Huangshan City, Anhwei Province	0.47613365	0.7375088	0.00423974	0.97794275
	182				0.46936353	0.7461651	0.00432030	0.97833401
92	183	117.9359 °	30.2279 °	Shuxi Village, Taoyuan Township, Huangshan District, Huangshan City, Anhwei Province	0.45325933	0.7047883	0.00000000	0.58866979
	184				0.43616782	0.7210173	0.00247106	0.59813220
93	185	118.081 °	32.2109 °	Huangli Village, Shi Pei Township, Quangjiao County, Chuzhou City, Anhwei Province	0.49904299	1.0826975	0.00267734	0.73254671
	186				0.51814115	1.0926591	0.00277718	0.73730052
94	187	118.0437 °	32.1113 °	Gangyang Village, The horse factory Township, Quangjiao County, Chuzhou City, Anhwei Province	0.51774382	1.1739475	0.00245778	0.57199393
	188				0.50328500	1.1762931	0.00235290	0.56795044
95	189	118.3654 °	32.5603 °	Revive the forest farm, Shunshan Township, Laian County, Chuzhou City, Anhwei Province	0.53159864	0.7180835	0.00281618	0.64726723
	190				0.52193695	0.7051537	0.00281887	0.65983524
96	191	118.5174 °	32.5636 °	Goutan Village, Zhangshan Township, Laian County, Chuzhou City, Anhwei Province	0.57160316	0.8259944	0.00256867	1.40455228
	192				0.56570086	0.8256642	0.00278377	1.39740983
97	193	110.969 °	22.3584 °	Bajiao Village, Guixi Township, Beichuan County, Mianyang City, Guangdong Province	0.45856968	1.2102675	0.00369707	0.43063588
	194				0.47125363	1.2507461	0.00365663	0.43690074
98	195	110.952 °	22.3502 °	Forestry institute, DongZhen Sub-District Office, Xinyi County-level City, Maoming City, Guangdong Province	0.30516140	0.5259123	0.00240341	0.55863262
	196				0.30785395	0.5787551	0.00255336	0.55453561
99	197	111.3805 °	22.3528 °	Yellow sand Village, Heshui Township, Maoming City, Guangdong Province	0.46837239	1.6986303	0.00391480	0.96742148
	198				0.47389386	1.710154	0.00400105	0.97043664
100	199	111.0295 °	22.0093 °	Soil domain Village, Changpo Township, Kochow County, Maoming City, Guangdong Province	0.38926103	1.2180431	0.00114910	0.48114589
	200				0.38875609	1.2677905	0.00134356	0.48045487
101	201	111.1819 °	22.1469 °	Guding Village, Guding Township, Kochow County, Maoming City, Guangdong Province	0.46171494	1.0971161	0.00259125	0.62721352
	202				0.46135882	1.1108236	0.00286436	0.62881083

102	203	113.1637°	24.7292°	Tangpen Village, Dongping Township, Ruyuan County, Shaoguan City, Guangdong Province	0.30469634	1.2867731	0.00255385	0.59262896
	204				0.30510171	1.3123412	0.00258669	0.58677220
103	205	113.3704°	24.8429°	Miaobei Village, One and six Township, Ruyuan County, Shaoguan City, Guangdong Province	0.40443278	1.0556494	0.00146219	0.33916963
	206				0.39749791	1.0507165	0.00152973	0.33157960
104	207	113.9556°	24.0127°	Changyin Village, Shatian Township, Xinfeng County, Shaoguan City, Guangdong Province	0.40857649	1.1931382	0.00343596	0.67610772
	208				0.41207290	1.1928994	0.00354446	0.66999583
105	209	114.5132°	24.1684°	Zhaixia Village, The Town Of Matou Township, Xinfeng County, Shaoguan City, Guangdong Province	0.41424752	1.1371983	0.00277718	0.45973596
	210				0.41604403	1.1748371	0.00284893	0.45561956
106	211	113.9076°	32.1508°	Chen Wan Village, Dong Guhe Township, Shihe District, Xinyang City, He'nan Province	0.46914327	0.7971005	0.00338560	0.65912584
	212				0.46464754	0.7188525	0.00339208	0.64778697
107	213	114.0033°	32.0853°	Huangwan Village, Ten three mile bridge Township, Shihe District, Xinyang City, He'nan Province	0.45129728	0.7249736	0.00341489	0.58022729
	214				0.45752071	0.7484909	0.00339256	0.57929533
108	215	115.6403°	31.9075°	Hanzhuang Village, Duanji Township, Gushi County, Xinyang City, He'nan Province	0.47940947	0.7594847	0.00283691	0.73078737
	216				0.47091483	0.7469515	0.00288199	0.73465098
109	217	115.7411°	31.8592°	Taiping Village, Wumiao Township, Gushi County, Xinyang City, He'nan Province	0.44152494	1.2786056	0.00207948	0.51845132
	218				0.43831904	1.2498798	0.00208149	0.51259750
110	219	113.269°	32.4283°	Gongzhuang Village, Huaiyuan Township, Tongbai County, Nanyang City, He'nan Province	0.42409916	0.4576533	0.00223838	0.38327129
	220				0.41880640	0.4626743	0.00227060	0.38194975
111	221	113.6755°	32.4922°	Maoji forest farm, Maoji Township, Tongbai County, Nanyang City, He'nan Province	0.40523852	1.1331941	0.00212459	0.56553982
	222				0.39202426	1.0970441	0.00223536	0.56160342
112	223	113.3515°	32.3868°	Miles east Village, Chengjiao Township, Tongbai County, Nanyang City, He'nan Province	0.40735413	1.1445159	0.00358862	0.63114993
	224				0.40540397	1.1518774	0.00348597	0.63355119
113	225	111.1255°	33.4565°	Garden guan Village, Xiping Township, Xixia County, Nanyang City, He'nan Province	0.33070674	0.4068354	0.00215175	0.41913076
	226				0.32925475	0.4192473	0.00221055	0.41681038
114	227	111.1617°	33.447°	Yuntai Village, Chongyang Township, Xixia County, Nanyang City, He'nan Province	0.38082578	0.8957045	0.00380944	0.63192908
	228				0.38270746	0.8900231	0.00377539	0.63205549
115	229	117.5342°	25.2141°	Hanmei Village, Luzhi Township, Zhangping	0.34261554	0.9411328	0.00280895	0.44296379

115	230	117.5342	25.2141	County-level City, Longyan City, Fujian Province	0.33258017	0.9000067	0.00275661	0.44461546
116	231	117.4585°	24.9686°	The May Day forest farm, Guidong Village, Guantian Township, Zhangping County-level City, Longyan City, Fujian Province	0.28943956	0.4769107	0.00248628	0.59203460
	232				0.29848183	0.4496973	0.00240499	0.58660061
117	233	117.3615°	25.6014°	Dongyang Village, Shuangyang Township, Zhangping County-level City, Longyan City, Fujian Province	0.38496541	0.9785239	0.00197278	0.80417599
	234				0.37917804	0.9452353	0.00198831	0.80015637
118	235	116.5161°	24.9453°	Fengkang Village, Lu feng she zu Township, Shanghang County, Longyan City, Fujian Province	0.35115935	1.2611172	0.00274299	0.72603175
	236				0.34825123	1.2561449	0.00274377	0.72354141
119	237	116.6068°	25.1514°	Baisha forest farm, Baisha Township, Shanghang County, Longyan City, Fujian Province	0.44963542	1.2344094	0.00246419	0.73697849
	238				0.45601112	1.2242598	0.00239892	0.72991610
120	239	118.5376°	26.9293°	Xiayang Village, Yushan Township, Jianou County-level City, Nanpin City, Fujian Province	0.35321713	1.6154021	0.00157623	0.46593772
	240				0.35853468	1.6145516	0.00141242	0.46584819
121	241	118.7386°	27.1723°	Taoyuan Village,Shuiyuan Township, Jianou County-level City, Nanpin City, Fujian Province	0.16944367	1.5593471	0.00176261	0.35053392
	242				0.17245574	1.5589699	0.00181250	0.34382221
122	243	117.6955°	27.0683°	WeiMin forest farm, WeiMin Village,WeiMin Township, Shaowu County-level City, Nanpin City, Fujian Province	0.40284520	1.0553728	0.00191069	0.49789930
	244				0.41284803	1.0526634	0.00184338	0.50301711
123	245	117.6955°	27.0683°	WeiMin forest farm, WeiMin Village,WeiMin Township, Shaowu County-level City, Nanpin City, Fujian Province	0.37510174	1.0396334	0.00202768	0.54380343
	246				0.38706347	1.0448692	0.00204460	0.54876735
124	247	117.3533°	27.3014°	Yanshan Village, Yanshan Township, Shaowu County-level City, Nanpin City, Fujian Province	0.52324674	1.1086984	0.00289743	0.75936129
	248				0.52613043	1.0629107	0.00291119	0.75315998
125	249	111.0611°	30.859°	Zhu Guwan Village, Letianxi Township, Yiling District, Yichang City, Hubei Province	0.48033790	0.8434243	0.00256094	0.49899337
	250				0.47737781	0.879702	0.00259676	0.50659555
126	251	111.4322°	30.9053°	Liu Guchong Village, Fenxiang Township, Yiling District, Yichang City, Hubei Province	0.45666279	0.7072893	0.00289186	0.61667063
	252				0.45861810	0.6877746	0.00288322	0.62492165
127	253	110.9718°	31.193°	Nanduihe Village, Shuiyue templeTownship, Xingshan County, Yichang City, Hubei Province	0.40075695	0.9055488	0.00279969	0.50150116
	254				0.40641167	0.9446282	0.00279761	0.50418935
128	255	110.7995°	31.32°	Shuimoxi Village, Huangliang Township,	0.45048115	1.3648745	0.00250810	0.90758949

[illegible]	256	[illegible]	[illegible]	Xingshan County, Yichang City, Hubei Province	0.45069609	1.4039437	0.00248017	0.90647660
129	257	114.5681°	31.0774°	Atealeaf an Village, Mier temple Township, Hongan County, Huanggan City, Hubei Province	0.44199454	0.778399	0.00236708	0.54055099
	258				0.44541191	0.7952786	0.00246546	0.54201867
130	259	114.6502°	31.4513°	GuFeng ridge Village, QiLiPing Township, Hongan County, Huanggan City, Hubei Province	0.50472580	0.9777499	0.00331945	1.07675443
	260				0.49948341	0.9831439	0.00340471	1.08046990
131	261	115.3435°	30.8678°	Dongcheng fan Village, Pinghu Township, Luotian County, Huanggan City, Hubei Province	0.43539238	1.6020297	0.00188427	0.61547719
	262				0.43659566	1.5391002	0.00187482	0.61285493
132	263	115.288°	30.8305°	Longhe Village, Three mile fan Township, Luotian County, Huanggan City, Hubei Province	0.47515706	1.1782766	0.00247840	0.78000183
	264				0.47865184	1.1720073	0.00267546	0.78167256
133	265	116.0657°	29.6711°	Chiang's shop, Qijia Township, Lushan District, Jiujiang City, Jiangxi Province	0.46341743	0.972066	0.00291157	0.59409133
	266				0.46858449	0.9504563	0.00291222	0.58560907
134	267	115.9339°	29.5801°	Saiyang Village, Saiyang Township, Lushan District, Jiujiang City, Jiangxi Province	0.45318051	0.7786453	0.00297685	0.56226860
	268				0.45225902	0.7492801	0.00300809	0.56800345
135	269	115.033°	29.4834°	Plantain Village, Full mouth Township, Wuning County, Jiujiang City, Jiangxi Province	0.47816941	1.2712571	0.00293940	0.90557602
	270				0.47678848	1.3220864	0.00296023	0.90381251
136	271	114.9945°	29.0986°	Fengyuan Village, Luoxi Township, Wuning County, Jiujiang City, Jiangxi Province	0.41231632	1.183587	0.00295075	0.89345035
	272				0.41819459	1.1972985	0.00296082	0.89934330
137	273	116.1702°	27.9733°	Tangnan Village, Gaoping Township, Linchuan District, Fuzhou City, Jiangxi Province	0.43984649	0.954465	0.00333092	0.75163294
	274				0.44535457	0.9639783	0.00329823	0.75276799
138	275	116.2402°	27.9771°	Security Village, Wenquan Township, Linchuan District, Fuzhou City, Jiangxi Province	0.49932137	1.3245269	0.00332233	0.73063492
	276				0.49447179	1.3050348	0.00345242	0.73105806
139	277	116.3068°	26.6734°	Xiaping Village, Xujiang Township, Guangchang County, Fuzhou City, Jiangxi Province	0.40103201	1.8326559	0.00355040	0.85791691
	278				0.40392205	1.8466715	0.00349129	0.86023291
140	279	116.3407°	26.7063°	Huixin Village, Chishui Township,Guangchang County, Fuzhou City, Jiangxi Province	0.36230448	1.4724103	0.00443610	1.00876152
	280				0.36863591	1.4773955	0.00442345	1.00199082

Table S2：The data of 55 ecological factors

Number	Name	Unit	Type
BIO 1～BIO 12	Monthly precipitation from January to December	mm	Successive type
BIO 13～BIO 24	Monthly average temperature from January to December	℃ ×10	Successive type
BIO 25	Annual average temperature	℃ ×10	Successive type
BIO 26	Mean day and night temperature difference	℃ ×10	Successive type
BIO 27	Isothermal property	1	Successive type
BIO 28	Standard deviation of seasonal variation in temperature	1	Successive type
BIO 29	Maximum temperature of warmest month	℃ ×10	Successive type
BIO 30	Minimum temperature of coldest month	℃ ×10	Successive type
BIO 31	Mean annual temperature range	℃ ×10	Successive type
BIO 32	Average temperature of most wet season	℃ ×10	Successive type
BIO 33	Average temperature of driest season	℃ ×10	Successive type
BIO 34	Average temperature of warmest season	℃ ×10	Successive type
BIO 35	Average temperature of coldest season	℃ ×10	Successive type
BIO 36	Average annual precipitation	mm	Successive type
BIO 37	Precipitation of most wet month	mm	Successive type
BIO 38	Precipitation of month	mm	Successive type
BIO 39	Coefficient of variation of seasonal precipitation	1	Successive type
BIO 40	Precipitation of most wet season	mm	Successive type
BIO 41	Precipitation of driest season	mm	Successive type
BIO 42	Precipitation of warmest season	mm	Successive type
BIO 43	Precipitation of coldest season	mm	Successive type
BIO 44	Topsoil pH value	1	Successive type

BIO 45	Cation exchange capacity of soil	cmol/kg	Successive type
BIO 46	Soil sediment concentration	%	Successive type
BIO 47	Soil clay content	%	Successive type
BIO 48	Subtype of soil（sym90）	1	Category type
BIO 49	Soil available water content	1	Category type
BIO 50	soil texture classification（USDA）	1	Category type
BIO 51	Topsoil organic carbon	%	Successive type
BIO 52	Elevation above sea level	m	Successive type
BIO 53	Slope	°	Successive type
BIO 54	Aspect	1	Category type
BIO 55	Sub category of vegetation	1	Category type

Note: climate data including precipitation of each month (BIO 1~12), average temperature of each month (BIO 13~24) and 19 integrated climate factors (BIO 25~43) were put out through interpolation of observed climate data from 1950 to 2000, with resolution ratio of 1 km. Soil data (BIO25~43) were based on *1:100 million soil map of the people's Republic of China*（1995) provided by the second national earth survey, with the applied soil taxonomy system of FAO-90. Terrain data include altitude with resolution ratio of 1 km (BIO 52), gradient (BIO 53) and aspect (BIO 54) Type of plants (BIO 55) were based on plant subgroup data in *vegetation map of the people's Republic of China (1:100 million)* published by *Institute of Botany, the Chinese Academy of Sciences*. Plotting scale were 1:1000,000 in nationwide range, with projection on WGS84 coordinate system, in Geotiff data format for all ecological factors above.
